# Computational and Molecular Dynamics Insights into the Antithrombotic Mechanism of Triterpenes Derived from *Melaleuca bracteata* var. Revolution Gold (Myrtaceae)

**DOI:** 10.3390/molecules31050848

**Published:** 2026-03-03

**Authors:** Patrick Appiah-Kubi, Foluso Oluwagbemiga Osunsanmi, Andrew Rowland Opoku, Ashona Singh

**Affiliations:** 1 Department of Chemistry, University of Pretoria, Hatfield, Pretoria 0002, South Africa; appiahpat@gmail.com; 2 Department of Biochemistry and Microbiology, University of Zululand, KwaDlangezwa, Empangeni 3886, South Africa; opokua@unizulu.ac.za; 3 Research Center, International School Development Organization (ISDO), Krugersdorp 1739, South Africa

**Keywords:** thrombin inhibition, 3β-acetoxybetulinic acid, betulinic acid, molecular docking, molecular dynamics simulation, binding energy, hydrogen bonding, natural product drug design

## Abstract

Background/Objectives: Thrombin, a serine protease central to coagulation and platelet activation, remains an important target for the development of safer and more effective antithrombotic agents. Naturally derived pentacyclic triterpenoids, such as betulinic acid and its acetylated derivatives, 3β-acetoxybetulinic acid, exhibit promising antiplatelet aggregation activity in validated in vitro and ex vivo assays; however, the molecular determinants underlying their direct thrombin inhibition remain unexplored. Results: Docking and MM/GBSA analyses revealed that Baa exhibits the strongest binding affinity (ΔG = −29.58 ± 2.97 kcal/mol), exceeding those of Ba (−20.94 ± 5.81 kcal/mol) and Asp (−18.87 ± 4.18 kcal/mol). Baa forms a highly persistent hydrogen bond with Trp96 (95.5% occupancy) and extensive hydrophobic contacts with Trp215, Leu99, Ile174, and Tyr60A residues defining thrombin’s aryl-binding pocket. MD trajectories demonstrated that Baa binding reduced solvent-accessible surface area (SASA) and residue fluctuations, indicating enhanced structural compaction and stability. In contrast, Ba exhibited weaker, transient hydrogen bonding, while Asp bound primarily near the catalytic triad. The triterpenes exhibit limited oral bioavailability, free PAINS alerts, favourable permeability and metabolic stability. Conclusions: Acetylation at C-3 (acetoxy substitution) substantially enhances thrombin binding via cooperative hydrogen bonding and van der Waals stabilisation, explaining the superior experimental inhibitory potency of Baa. These findings provide a mechanistic framework for structure-guided optimisation of triterpenoid-based thrombin inhibitors and support their further experimental development. Methods: In this study, molecular docking, molecular dynamics (MD) simulations (400 ns), and MM/GBSA free energy analyses were employed to elucidate the binding mechanisms of 3β-acetoxybetulinic acid (Baa), betulinic acid (Ba), and aspirin (Asp) within the thrombin receptor active site. The simulations were explicitly grounded in previously reported chromogenic antithrombin assays and platelet aggregation studies and were designed to mechanistically rationalise the experimentally observed inhibitory potency.

## 1. Introduction

Cardiovascular diseases remain the leading global cause of death [1]. Thrombosis is a major driver of the global cardiovascular disease burden and the leading cause of myocardial infarction, strokes, and venous thromboembolism. Venous thromboembolism, comprising pulmonary embolism and deep vein thrombosis, affects millions globally and remains a significant cause of morbidity and mortality [2]. Deep vein thrombosis (DVT) is characterised by the formation of a blood clot within the deep veins, most commonly in the legs. When a fragment of the clot breaks off, travels through the bloodstream, and gets trapped in the lungs, it causes a pulmonary embolism (PE)—a potentially fatal condition [3,4].

Thrombus formation is a multifactorial physiological process influenced by alterations in the vascular wall, blood flow, and blood composition. The fundamental processes involve platelet adhesion and aggregation at sites of endothelial injury, and activation of the coagulation cascade [5]. At the onset of vascular injury, platelets adhere to the exposed subendothelial matrix proteins through interactions between von Willebrand factor (vWF) and the platelet receptor glycoprotein (GP) Ibα-V-IX complex [6]. This initial adhesion triggers platelet activation, morphological change, and granule secretion, leading to fibrinogen binding to integrin αIIbβ3 and subsequent platelet aggregation. Concurrently, the coagulation cascade initiates through tissue factor binding to factor VII, leading to sequential activation of thrombin, factor X (FXa), factor IX (FIXa), and factor XI (FXIa), with cofactors V (FV) and VIII (FVIII) amplifying the response [7,8]. The resultant propagation of thrombin generation drives fibrin formation, stabilising the platelet plug and promoting thrombus development.

**Figure 1:**
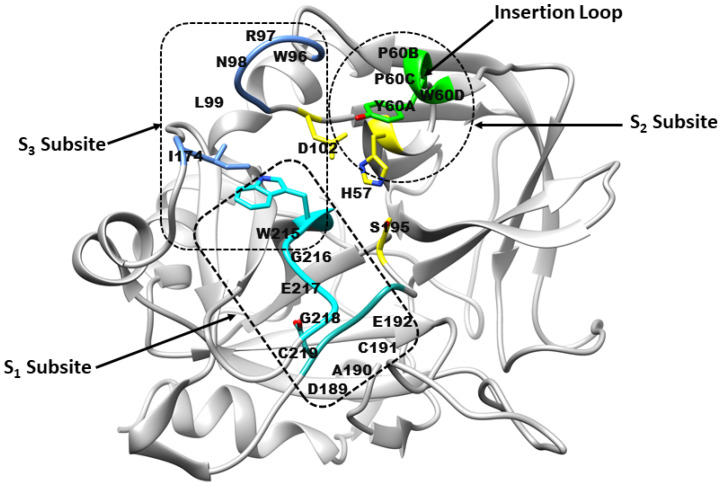
Architecture of the thrombin receptor binding site. Subsite S1 is formed by residues from the VSWGEGC motif (cyan) and the DACE motif (light sea green). Subsite S2 comprises His57 and residues Tyr60A–Trp60D (green), while subsite S3 consists of residues from the WRENL motif (cornflower blue). The catalytic triad residues His57, Ser195, and Asp102 are highlighted in yellow. (Figure created by the authors, based on concepts from refs. [9,10]).

The thrombin binding site comprises three subsites (Figure 1): S1, S2, and S3 [9]. The S1 subsite forms a large cavity, partially defined by residues from the VSWGEGC (Val213–Cys219) and DACE (Asp189–Glu192) motifs. Backbone atoms from these regions, together with the side chain of Val213, shape the pocket architecture, while Asp189 contributes a negatively charged environment. A stabilising disulfide bond between Cys191 and Cys219 further reinforces the S1 subsite. The S2 subsite is delineated by residues from the YPPW insertion loop (Tyr60A-Pro60B-Pro60C-Trp60D), together with His57 of the catalytic triad (His57, Ser195 and Asp102) [10]. Trp215 and Gly216 form the opposite boundary from the VSWGEGC motif. The S3 subsite (aryl-binding pocket) constitutes a large, solvent-exposed pocket with a pronounced amphipathic character. One boundary is formed by the WRENL motif (Trp96-Arg97-Glu97A-Asn98–Leu99), where backbone carbonyl groups and the Leu99 side chain create a hydrophilic surface, complemented by Tyr60A. The opposing boundary is defined by hydrophobic residues Ile174, Trp215, and Glu217 [9].

Therapeutic strategies targeting thrombosis, particularly antithrombotic, anticoagulant, and antiplatelet agents, have emerged as a key focus in antithrombotic drug discovery and molecular modelling studies. Current thrombin inhibitors are broadly categorised into direct and indirect agents based on their mechanism of action. Direct thrombin inhibitors (DTIs) bind directly to the active site of thrombin, thereby inhibiting fibrinogen cleavage and subsequent thrombus formation. Both parenteral DTIs, including lepirudin, desirudin, bivalirudin, and argatroban, as well as orally administered agents such as dabigatran, are widely used for the prophylaxis and treatment of venous thromboembolism (VTE) and for the prevention of thromboembolic complications in patients with, or at risk of, heparin-induced thrombocytopenia (HIT) [11,12,13]. In contrast, indirect inhibitors, including warfarin, heparin, and fondaparinux, act by inhibiting thrombin or factor Xa via antithrombin [11,14,15]. Despite their clinical utility, several limitations, such as bleeding complications, gastrointestinal ulceration, heparin-induced thrombocytopenia, purpura, and the need for frequent monitoring, underscore the need for alternative therapeutics [16,17]. Given the critical balance between efficacy and safety in drug development, there is an urgent need for cardiovascular therapeutics that minimise bleeding risk while maintaining effectiveness [18].

The limitations of existing antithrombotic agents have prompted growing interest in identifying plant-derived bioactive compounds with safer pharmacological profiles and multifunctional activity against thrombosis and platelet aggregation. Medicinal plants represent a promising alternative, owing to their accessibility, affordability, and perceived safety [19,20]. Recent studies have demonstrated antithrombotic, antiplatelet, and anticoagulant activities of several plant-derived bioactive compounds from *Campomanesia xanthocarpa*, *Ophiopogon japonicas*, *Dendropanax morbifera*, *Melaleuca bracteata* and *Panax notoginseng* [20,21,22,23,24].

**Figure 2:**
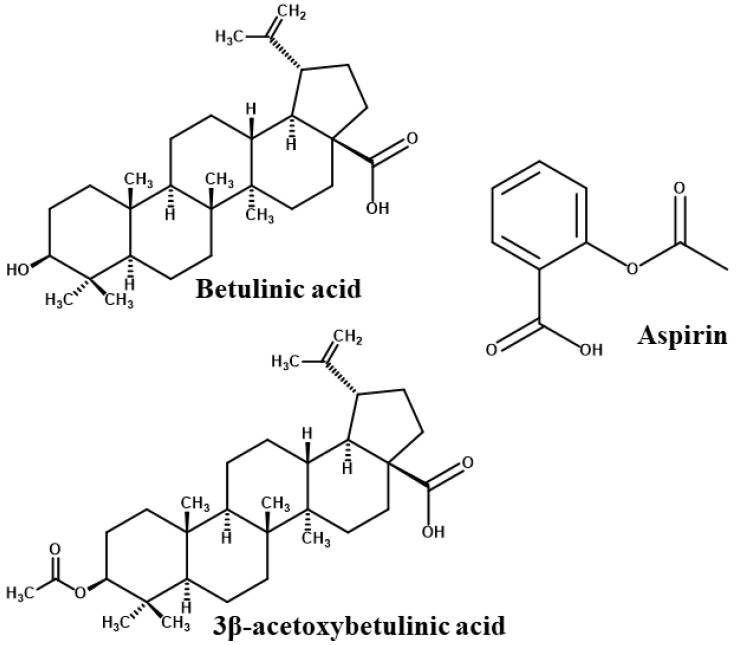
2D chemical structures of the studied compounds [26].

Betulinic acid (BA), a naturally occurring pentacyclic triterpenoid, and its derivative 3β-acetoxybetulinic acid (BAA), isolated and synthesised from *Melaleuca bracteata* ‘Revolution Gold’, have recently demonstrated promising antithrombotic, anticoagulant, and antiplatelet properties [25,26] (Figure 2). Both compounds significantly inhibited thrombin activity and thrombin-induced platelet aggregation in a dose-dependent manner. Notably, 3β-acetoxybetulinic acid exhibited a more potent inhibitory effect (IC_50_ = 1.10 ± 0.03 mg/mL) than betulinic acid (2.36 ± 0.09 mg/mL) and aspirin (2.65 ± 0.01 mg/mL), while exerting minimal effects on bleeding time compared to aspirin [25]. These findings suggest that acetylation may enhance the bioactivity and selectivity of 3β-acetoxybetulinic acid toward thrombin inhibition, highlighting the potential of these natural triterpenoids as safer antithrombotic agents. Despite their pharmacological potential, the atomistic mechanisms underlying the interactions of betulinic acid and 3β-acetoxybetulinic acid with thrombin remain unexplored.

Insights into their binding modes, conformational dynamics, pharmacokinetics, and energetic stability are essential for elucidating the structure–activity relationships underlying their observed biological efficacy. Molecular modelling provides a robust framework for investigating bioactive molecules and their interaction mechanisms, facilitating the identification of key molecular determinants underlying enhanced bioactivity. The present study employs molecular docking and molecular dynamics (MD) simulations not as independent biological validation, but as a mechanistic tool to explain and contextualise existing experimental data [25]. By mapping the binding interactions, conformational stability, and free-energy profiles of betulinic acid and 3β-acetoxybetulinic acid in complex with thrombin to known inhibitory outcomes. Comparative analysis of the systems provided insight into how specific molecular features of the inhibitors contribute to binding affinity through interactions with distinct subsites of the thrombin active site. By integrating computational and experimental evidence, this work seeks to provide a coherent, atomic-level mechanistic understanding, offering a foundation for structural modifications and the rational design of novel, naturally derived thrombin inhibitors with improved efficacy and safety.

## 2. Results

### 2.1. Molecular Docking of Ligands with Thrombin Receptor

**Figure 3:**
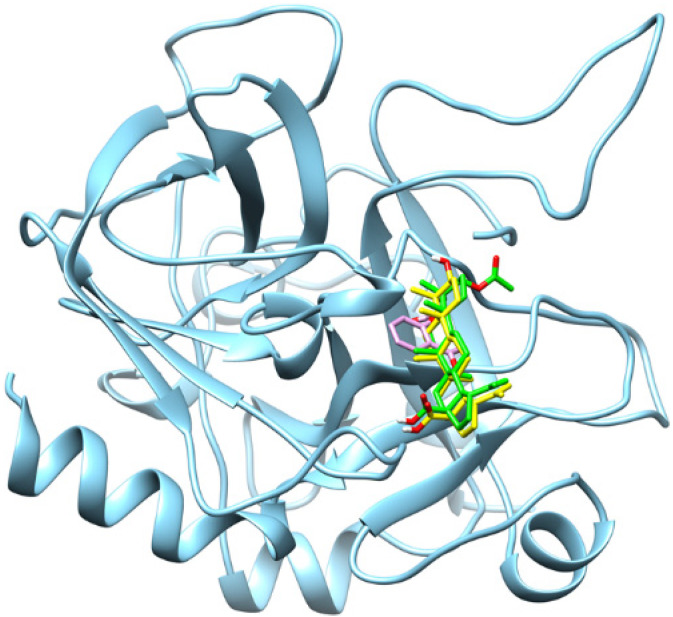
Superimposition of the binding orientations of the docked complexes, showing the thrombin receptor in cyan, 3β-acetoxybetulinic acid in green, betulinic acid in yellow, and aspirin in magenta within the thrombin receptor binding site.

Molecular docking analysis was conducted to characterise the binding interactions between thrombin and the selected ligands, with the resulting docking scores and key interacting residues summarised in Table 1. The docking analysis revealed distinct binding affinities and interaction profiles for 3β-acetoxybetulinic acid (Baa), betulinic acid (Ba), and aspirin (Asp) within the thrombin receptor active site (Figure 3). Among the three compounds, 3β-acetoxybetulinic acid exhibited the highest binding affinity, with an XP GScore of −8.38 kcal/mol and an IFD score of −635.69, followed closely by betulinic acid (−8.22 kcal/mol and −635.66). Aspirin showed comparatively weaker binding (−5.49 kcal/mol and −631.05), indicating less favourable interaction with the thrombin active site.

**Figure 4:**
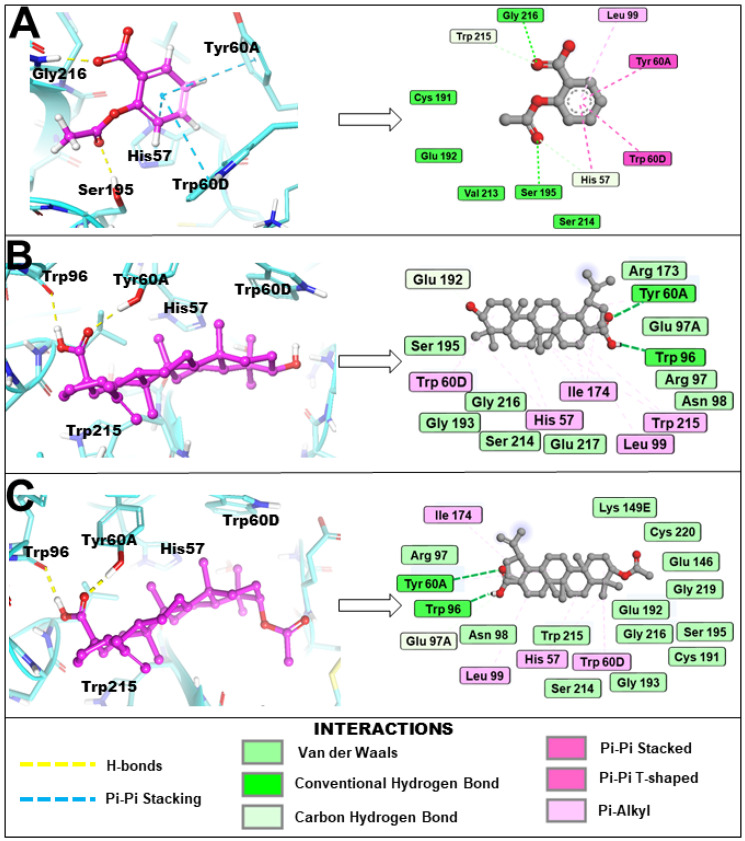
Binding orientation and key binding interaction profile of (**A**) aspirin, (**B**) betulinic acid and (**C**) 3β-acetoxybetulinic acid within the active site (cyan) of the thrombin receptor revealed by induced fit molecular docking.

The binding orientation and key binding interaction profile are presented in Figure 4. Hydrogen bonding analysis revealed that both 3β-acetoxybetulinic acid and betulinic acid formed hydrogen bonds primarily with Trp96 and Tyr60A, residues situated near the entrance of the thrombin binding pocket, contributing to initial ligand anchoring and orientation. In contrast, aspirin formed hydrogen bonds with Gly216 and Ser195, residues located within the catalytic pocket, consistent with its smaller size and higher polarity. Hydrophobic contacts dominated the stabilisation of 3β-acetoxybetulinic acid and betulinic acid within the binding cleft, involving Leu99, Trp96, Tyr60A, Val213, Trp215, and Cys191, which are critical residues forming the substrate recognition region of thrombin. Both ligands also interacted with Pro60C, Trp60D, and Ile174, suggesting extensive van der Waals stabilisation within the S1–S3 subsites. Notably, 3β-acetoxybetulinic acid exhibited additional interactions with Cys220, which may contribute to its slightly enhanced binding affinity relative to betulinic acid.

Aspirin displayed a different interaction pattern, characterised by both hydrophobic and π–π stacking interactions involving His57, Tyr60A, and Trp60D, consistent with its ability to engage the catalytic triad region. However, the lower docking score and fewer hydrophobic contacts suggest a less stable complex than the triterpenoid derivatives. Collectively, these results indicate that 3β-acetoxybetulinic acid achieves the most stable binding conformation within the thrombin receptor through an optimal balance of hydrogen bonding and hydrophobic interactions.

### 2.2. ADME and Physicochemical Properties

The predicted physicochemical and pharmacokinetic profiles of 3β-acetoxybetulinic acid, betulinic acid, and aspirin are presented in Table 2. Consistent with their bulky pentacyclic scaffolds, 3β-acetoxybetulinic acid and betulinic acid possessed higher molecular weights (498.37 g/mol and 456.36 g/mol, respectively) compared to aspirin (180.04 g/mol). Their moderate polar surface areas (63.6 and 57.5 Å^2^) and high molar refractivity values suggest balanced polarity and a strong van der Waals interaction potential. However, their high lipophilicity (Log P = 4.65 and 4.25) and poor aqueous solubility (Log S ≈ −4.90) may limit gastrointestinal absorption and bioavailability relative to aspirin (Log P = 1.17; Log S = −1.55).

In silico ADME predictions indicated low gastrointestinal absorption and poor blood–brain barrier (BBB) permeation for both triterpenoids, whereas aspirin showed high absorption and BBB permeation, consistent with its well-characterised pharmacokinetics. Among the compounds, only 3β-acetoxybetulinic acid showed inhibitory activity against CYP2C9, suggesting a potential influence on hepatic metabolism. None were predicted to be P-glycoprotein substrates, indicating minimal risk of efflux-mediated clearance. The triterpenoids also exhibited superior skin permeability (Log Kp ≈ −3.1 cm/s) relative to aspirin (−6.55 cm/s), highlighting their potential suitability for transdermal delivery.

All compounds were free of PAINS alerts, indicating low risk of assay interference. Their synthetic accessibility scores classified them as readily synthesizable, while the high natural product likeness values (NP ≈ 3.0) for the triterpenoids underscore their structural diversity and drug-like natural origins. Overall, the data suggest that although 3β-acetoxybetulinic acid and betulinic acid may exhibit limited oral bioavailability, their favourable permeability, metabolic stability, and structural features make them promising leads for further development, particularly through optimisation of formulation or delivery strategies.

### 2.3. Structural Stability and Conformational Dynamics

**Figure 5:**
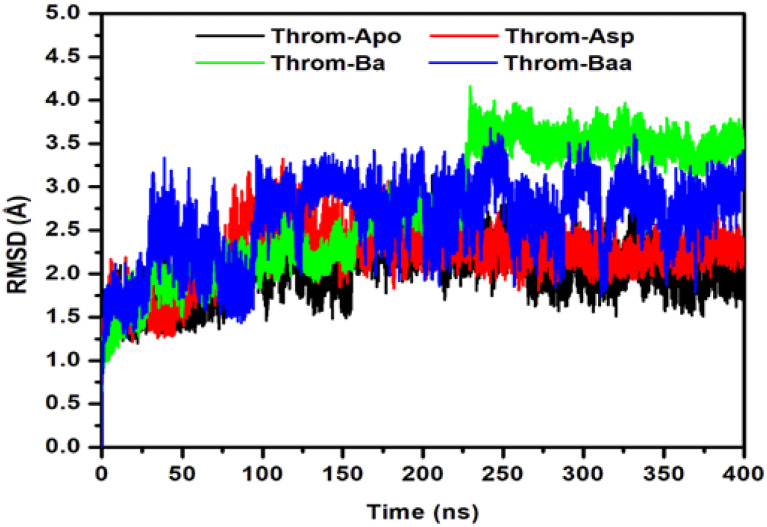
Root Mean Square Deviation (RMSD) of the thrombin receptor backbone during 400 ns MD simulations for the Apo, aspirin (Asp), betulinic acid (Ba), and 3β-acetoxybetulinic acid (Baa) systems.

The conformational convergence of the four simulated systems was first evaluated using the root-mean-square deviation (RMSD) of the protein backbone over 400 ns of molecular dynamics (MD) simulations to assess the structural stability of the protein-ligand complexes (Figure 5). The apo protein (Throm-Apo) equilibrated with a mean backbone RMSD of 2.02 ± 0.31 Å, indicating a well-retained fold. The Aspirin complex (Throm-Asp) showed a modest increase (2.23 ± 0.32 Å) relative to the apo, consistent with a small ligand-induced perturbation of the global fold. In contrast, the betulinic acid complex (Throm-Ba) and the 3β-acetoxybetulinic acid complex (Throm-Baa) showed greater deviations, indicating more pronounced structural rearrangements and greater dynamic heterogeneity upon binding. Throm-Baa displays an elevated RMSD from early in the simulation and fluctuates around ~2.5–3.2 Å, giving a mean RMSD of 2.63 ± 0.46 Å. Throm-Ba shows the most significant effect, with an abrupt upward shift at ≈230 ns and a sustained plateau of higher RMSD values thereafter (fluctuating near ~3.2–3.8 Å), yielding the largest mean and dispersion (2.78 ± 0.73 Å).

Taken together, these results suggest two distinct ligand behaviours: (i) Asp, which binds without substantially disturbing the overall backbone fold, and (ii) Ba/Baa, which are associated with larger-scale backbone rearrangements from its native state. The significant standard deviation for Throm–Ba may imply either switching between multiple conformational substates or sampling of one or more higher-RMSD conformers for substantial fractions of the trajectory.

**Figure 6:**
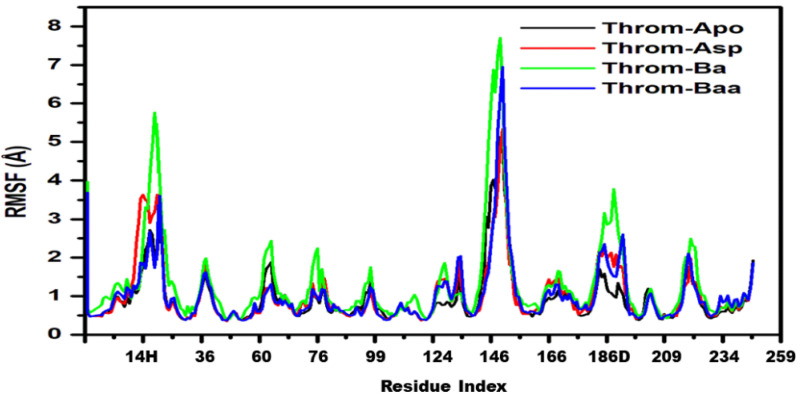
Root mean square fluctuation (RMSF) of the thrombin receptor backbone for Throm-Apo (black), Throm-Asp (red), Throm-Ba (green) and Throm-Baa (blue) over the 400 ns MD trajectories.

To characterise the intrinsic residue conformational dynamics of Throm-Apo, Throm-Asp, Throm-Ba, and Throm-Baa in response to ligand binding, root mean square fluctuation (RMSF) analysis was conducted on backbone atoms. The RMSF profiles (Figure 6) revealed comparable global fluctuation patterns across all four systems, reflecting overall structural stability of the thrombin receptor. Higher flexibility was primarily localised to the N-terminal region and loop segments spanning residues 14G-14L, 30–40, 145–150, and 185–195, suggesting intrinsic mobility. Among the ligand-bound forms, Throm-Ba exhibited the highest fluctuation, particularly near residue ~145–147, which may indicate that betulinic acid binding induces localised dynamic flexibility. Conversely, Throm-Asp and Throm-Baa generally displayed reduced residue fluctuations upon ligand binding, indicative of enhanced conformational rigidity and stabilisation. These findings imply that 3β-acetoxybetulinic acid and aspirin may confer increased structural stability to the thrombin receptor upon binding.

**Figure 7:**
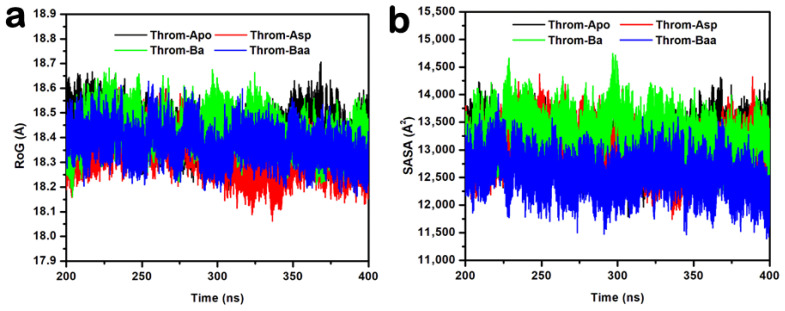
(**a**) Radius of gyration (RoG) and (**b**) solvent accessible surface area (SASA) plots for the thrombin receptor during the equilibrated last 200 ns of the 400 ns MD simulations.

The Radius of Gyration (RoG) quantifies a protein’s overall compactness and provides insight into its conformational stability. Proteins with elevated RoG values adopt expanded conformations that are generally less stable, whereas those with lower RoG values exhibit tighter, more stable packing. The studied systems remained relatively stable throughout the equilibrated 200 ns portion across all system trajectories, indicating minimal large-scale structural changes (Figure 7a). Quantitatively, the mean RoG values were comparable (Apo 18.43 ± 0.06 Å, Asp 18.33 ± 0.07 Å, Ba 18.43 ± 0.07 Å, Baa 18.37 ± 0.06 Å) across systems, indicating overall structural stability of the thrombin receptor during the last 200 ns. The slightly lower RoG for the aspirin complex suggests a marginal increase in compactness upon aspirin binding. At the same time, the minimal variations across all systems imply that ligand association did not significantly alter the receptor’s global conformation. Betulinic acid and 3β-acetoxybetulinic acid show nearly identical RoG with a 0.06 Å difference that lies within the reported standard deviations. This means that both complexes have essentially the same global compactness; 3β-acetoxybetulinic acid is nominally, but not significantly, more compact than betulinic acid. The slight difference could reflect subtle local rearrangements or tighter packing around the ligand in 3β-acetoxybetulinic acid, but it is not evidence of a global conformational change.

The Solvent Accessible Surface Area (SASA) analysis provides insight into the degree of protein surface exposure to solvent and can indicate conformational changes during molecular dynamics simulations. The SASA plots for the last stable 200 ns (Figure 7b) show comparable values with minor fluctuations around stable averages for Apo (13,119.34 ± 286.94 Å^2^), aspirin (13,019.54 ± 314.89 Å^2^), and betulinic acid (13,285.60 ± 312.94 Å^2^), suggesting similar levels of solvent exposure and structural compactness. In contrast, the 3β-acetoxybetulinic acid complex exhibited a markedly lower SASA (12,556.45 ± 292.49 Å^2^), implying a more compact structure and reduced solvent accessibility upon ligand binding. This observation suggests that 3β-acetoxybetulinic acid may enhance the overall structural stabilisation of the thrombin receptor by promoting tighter molecular packing.

### 2.4. Hydrogen Bond Interaction and Occupancy Analysis

**Figure 8:**
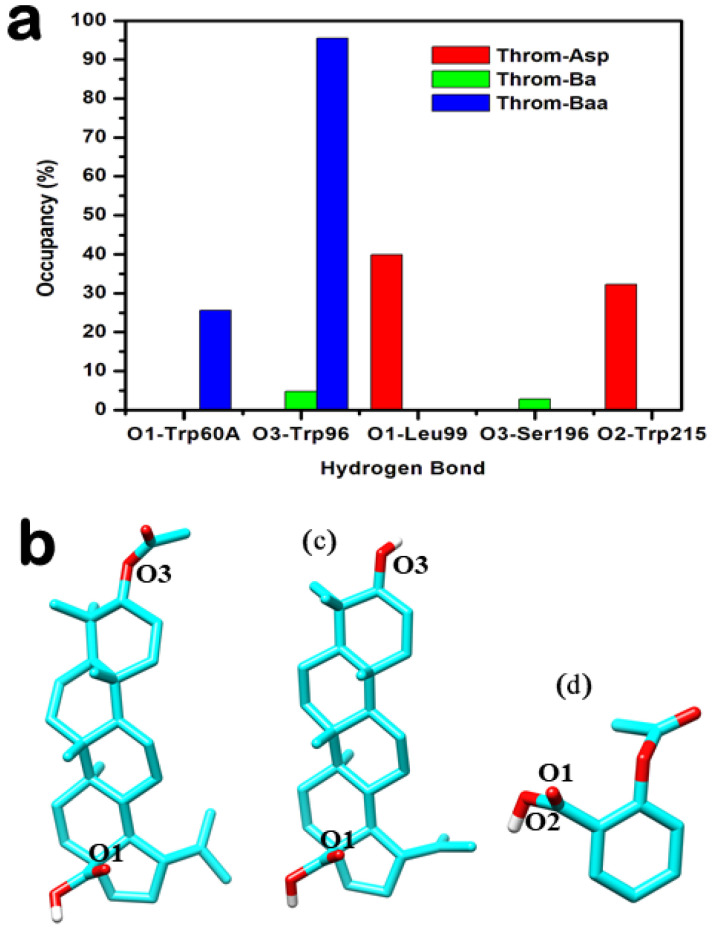
Hydrogen bond interaction analysis between the three inhibitors and the thrombin receptor derived from molecular dynamics (MD) trajectories. (**a**) Overview of hydrogen bond interactions observed throughout the MD simulations. (**b**–**d**) Structural representations showing the positions of key atoms participating in hydrogen bonding for 3β-acetoxybetulinic acid (**b**), betulinic acid (**c**), and aspirin (**d**).

Hydrogen bonding is crucial for protein-ligand complex binding specificity and stability during MD simulations [27]. To characterise these interactions, hydrogen bond occupancies were analysed over the 400 ns MD trajectories for 3β-acetoxybetulinic acid, betulinic acid, and aspirin bound to the thrombin receptor. The analysis revealed distinct hydrogen-bonding profiles among the ligands (Table 3 and Figure 8).

3β-acetoxybetulinic acid exhibits a highly persistent hydrogen bond with Trp96-O with an occupancy of 95.51%, alongside a secondary bond of 25.64% occupancy with Tyr60A-OH. The presence of the acetoxy group at C-3 appears critical for stabilising this interaction, which is absent or significantly weaker in the parent compound. In contrast, betulinic acid shows markedly lower occupancies across all interactions, with its highest at only 4.73% (Trp96…Lig248). Multiple transient contacts (e.g., Ser195, Trp148, and Gly216) suggest weak and dynamic binding, indicating that removal of the acetoxy moiety compromises stable engagement with thrombin. Aspirin, despite its smaller size, forms moderately stable H-bonds with Leu99-N (39.88%) and Trp215-O (32.26%), but does not interact with Trp96, suggesting a different binding orientation and site. Notably, the high-occupancy interaction with Trp96 observed only in the 3β-acetoxybetulinic acid complex points to a synergistic role of structural modification in enhancing binding stability. This residue may serve as a key anchoring point for ligands designed to exploit thrombin’s active site.

### 2.5. Number of Hydrogen Bond Analysis

**Figure 9:**
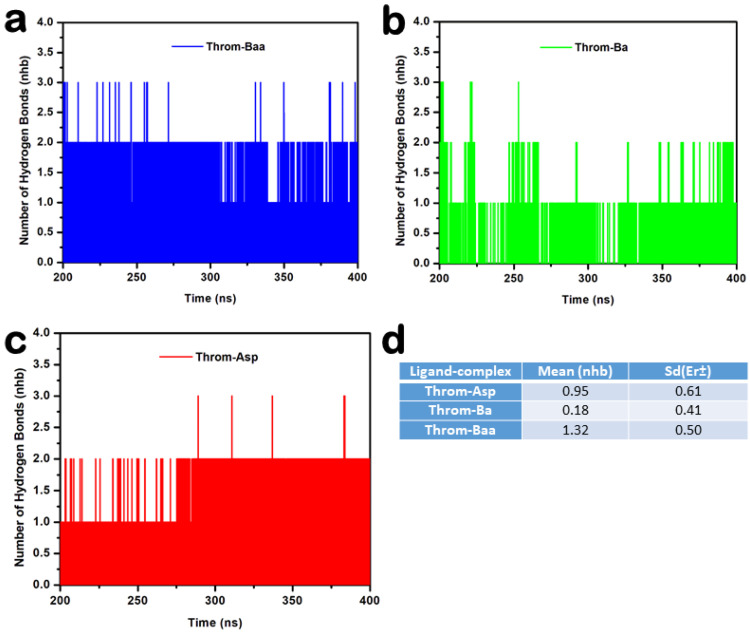
Hydrogen bond analysis of thrombin–ligand complexes during 200–400 ns molecular dynamics simulation. (**a**) 3β-acetoxybetulinic acid (Throm–Baa), (**b**) Betulinic acid (Throm–Ba), and (**c**) Aspirin (Throm–Asp) complexes showing the number of hydrogen bonds (nhb) formed over time. (**d**) Average hydrogen bond count (Mean ± SD) for each complex.

Hydrogen bonds were monitored to assess the stability of thrombin–ligand interactions during the last 200 ns molecular dynamics simulations (Figure 8a–d). The 3β-acetoxybetulinic acid complex maintained the highest average hydrogen bond count of 1.32 ± 0.50, forming one to three hydrogen bonds consistently with minimal fluctuation, indicating strong and stable binding (Figure 9a). In contrast, the betulinic acid complex exhibited weak and transient hydrogen bonding, with a mean hydrogen bond count of 0.18 ± 0.41, typically maintaining fewer than 1 hydrogen bond throughout the simulation, reflecting poor interaction stability (Figure 9b). The aspirin complex showed intermediate behaviour, keeping one to two hydrogen bonds intermittently (Figure 9c), with an average hydrogen bond count of 0.95 ± 0.61. Taken together, the hydrogen bond occupancy and interaction analyses indicate that 3β-acetoxybetulinic acid forms the most persistent hydrogen bonding network with thrombin. This suggests greater complex stability and binding affinity than those of betulinic acid and aspirin.

### 2.6. Binding Free Energy and Hotspot Residue Analysis

To gain mechanistic insight into binding affinity, the binding free energies (ΔG) of the ligand–protein complexes were quantitatively evaluated using the MM/GBSA approach. This analysis allowed decomposition of the total binding free energy (ΔG) into individual energetic contributions, thereby elucidating the specific energetic contributions of the key interactions governing molecular recognition, as detailed in Table 4. The calculated binding free energies (ΔG) revealed that 3β-acetoxybetulinic acid was the most potent binder (−29.58 ± 2.97 kcal/mol), followed by Betulinic acid (−20.94 ± 5.81 kcal/mol). In contrast, the Aspirin complex displayed a less favourable binding energy of −18.87 ± 4.18 kcal/mol. Enhanced binding of 3β-acetoxybetulinic acid is primarily driven by strong van der Waals interactions (−35.26 ± 2.80 kcal/mol), exceeding those of betulinic acid (−30.65 ± 7.10 kcal/mol) and aspirin (−22.62 ± 2.62 kcal/mol), indicating a more extensive or complementary hydrophobic interface. Electrostatic contributions (−11.30 ± 4.14 kcal/mol) further stabilised the complex, surpassing those of betulinic acid (−5.49 ± 5.51 kcal/mol). While aspirin showed stronger electrostatics (−20.02 ± 5.00 kcal/mol), this advantage was offset by higher desolvation costs (23.76 ± 3.43 kcal/mol).

To identify residues critical for ligand binding, per-residue decomposition of the binding free energy was carried out. Hotspots were defined as residues exhibiting highly favourable interaction energies (ΔG_Total_ > 1.00 kcal/mol, highlighted in green) with 3β-acetoxybetulinic acid, betulinic acid, and aspirin (Table 5). Among the studied ligands, 3β-acetoxybetulinic acid established the most favourable interactions. Six hotspot residues, primarily hydrophobic and aromatic, were key to its stability: Trp215 (−2.03 ± 0.48 kcal/mol), Ile174 (−1.67 ± 0.36 kcal/mol), Trp96 (−1.65 ± 0.47 kcal/mol), Leu99 (−1.57 ± 0.35 kcal/mol), Tyr60A (−1.31 ± 1.07 kcal/mol), and Asn98 (−1.02 ± 0.29 kcal/mol). This suggests that π–π stacking and van der Waals forces are the dominant stabilising interactions, likely enhanced by the acetoxy moiety, which improves conformational and hydrophobic complementarity. In contrast, betulinic acid showed a similar but weaker binding profile, with notable contributions from Trp60D (−1.60 ± 0.98 kcal/mol) and Trp215 (−1.12 ± 0.74 kcal/mol), explaining its lower overall binding energy. Aspirin, on the other hand, exhibited fewer stabilising interactions compared to 3β-acetoxybetulinic acid. The most significant energetic contributions for Aspirin were observed for Leu99 (−2.00 ± 0.44 kcal/mol), Ile174 (−1.84 ± 0.47 kcal/mol), Trp215 (−1.73 ± 1.32 kcal/mol), and Asn98 (−1.45 ± 0.38 kcal/mol). These interactions, primarily involving localised hydrogen bonds and limited hydrophobic contacts, are consistent with aspirin’s smaller molecular size and reduced surface complementarity relative to 3β-acetoxybetulinic acid. Trp96 plays a critical role in the enhanced inhibitory activity of 3β-acetoxybetulinic acid, showing a unique and favourable per-residue energy contribution (−1.65 ± 0.47 kcal/mol) exclusive to this complex.

## 3. Discussion

This study integrates validated experimental observations with atomistic simulations to elucidate the molecular mechanisms underlying the thrombin inhibitory activity of betulinic acid derivatives. Previous experimental work demonstrated that 3β-acetoxybetulinic acid exhibits significantly greater antithrombin and antiplatelet activity than betulinic acid and aspirin, yet the structural basis for this enhancement remained unexplained. Recent progress in drug discovery has increasingly relied on molecular dynamics (MD) simulations to explore the conformational behaviour of proteins and other biomolecules at the atomic level. This approach has profoundly enhanced the rational design of small molecules, peptides, and proteins, while deepening insight into the structural mechanisms underlying various diseases [28,29].

Molecular docking and molecular dynamics (MD) simulations provided a comprehensive understanding of how structural variations in betulinic acid derivatives influence thrombin inhibition. Our findings demonstrate that 3β-acetoxybetulinic acid (Baa) exhibits the most favourable interaction profile with thrombin, followed by betulinic acid (Ba) and aspirin (Asp). The calculated binding free energies (ΔG) revealed that 3β-acetoxybetulinic acid was the most potent binder (−29.58 ± 2.97 kcal/mol), followed by Betulinic acid (−20.94 ± 5.81 kcal/mol). In contrast, the Aspirin complex displayed a less favourable binding energy of −18.87 ± 4.18 kcal/mol. The MM/GBSA binding free energy decomposition confirmed that van der Waals forces are the principal stabilising component (−35.26 ± 2.80 kcal/mol for Baa), exceeding those of Ba and Asp. This dominance of dispersion interactions is characteristic of hydrophobic inhibitors within thrombin’s aryl pocket (S_3_ subsite) [9]. The computational binding energy estimation was consistent with the experimental findings, wherein 3β-acetoxybetulinic acid demonstrated a more potent inhibitory effect (IC_50_ = 1.10 ± 0.03 mg/mL) than betulinic acid (2.36 ± 0.09 mg/mL) and aspirin (2.65 ± 0.01 mg/mL) [25]. The superior binding affinity of Baa is attributable to the presence of the acetoxy group at the C-3 position, which enhances hydrogen bonding and hydrophobic complementarity within thrombin’s active site. This computational trend closely correlates with previously reported experimental inhibition data, in which Baa showed more vigorous antithrombotic activity than Ba and aspirin [25].

These results indicate that the triterpene scaffold acts as a hydrophobic anchoring core, while polar substituents modulate binding affinity. Accordingly, future optimization should retain the pentacyclic backbone, introduce polar functional groups at solvent-exposed positions to strengthen electrostatic interactions, and avoid steric modifications that disrupt pocket complementarity. MM/GBSA energy decomposition further identified key stabilizing residues and interaction hotspots; notably, Trp96 plays a critical role in the enhanced inhibitory activity of 3β-acetoxybetulinic acid. Overall, the MM/GBSA analysis provides a structure–activity framework for the semi-synthetic design of more potent antithrombotic agents.

The molecular docking and per-residue decomposition analyses revealed that Baa establishes persistent interactions with key hydrophobic and aromatic residues—Trp215, Trp96, Leu99, Ile174, Tyr60A, and Asn98—which define the aryl-binding pocket (S_3_ subsite) of thrombin. The acetoxy substitution at C-3 facilitates the formation of a highly stable hydrogen bond with Trp96 (occupancy = 95.5%), a feature absent in Ba. The acetoxy moiety thus serves as a structural determinant for the orientation and stabilisation of Baa within the active pocket. This aligns with prior structural studies demonstrating the importance of aromatic residues in thrombin’s substrate recognition and inhibitor binding [30]. The predominance of van der Waals and hydrophobic interactions in the Baa–thrombin complex indicates that hydrophobic complementarity drives complex stabilisation, consistent with the reported binding modes of other antithrombotic agents [9,30]. By contrast, aspirin primarily engages in polar interactions within the catalytic triad (His57, Ser195, and Asp102), reflecting its smaller size and greater polarity. The weaker MM/GBSA binding energy of aspirin (−18.87 ± 4.18 kcal/mol) supports its limited capacity to form extensive hydrophobic networks within thrombin’s binding cleft.

To ensure adequate conformational sampling, we evaluated the convergence behavior of the 400 ns molecular dynamics trajectory rather than relying solely on simulation length. Backbone RMSD, radius of gyration, and total interaction energy profiles reach equilibrium plateaus and remain stationary during the final segment of the simulation, while residue-level RMSF values show only localized fluctuations consistent with thermal motion. Thus, the trajectory is sufficient to characterize the stability and interaction pattern of the receptor–ligand complex. We acknowledge that multiple independent replicas are generally desirable for exhaustive exploration of the global conformational landscape and improved statistical averaging. The demonstrated convergence, therefore, indicates that the long-timescale single trajectory adequately captures the relevant bound conformational ensemble for the conclusions drawn.

The RMSD and RMSF analyses revealed that Ba and Baa binding induced greater backbone deviations (mean RMSD ≈ 2.6–2.8 Å) than Asp or the apo form, suggesting localised structural rearrangements upon triterpenoid binding. Elevated RMSD in the presence of Baa likely reflects adaptive loop movements required to accommodate its bulky scaffold, rather than global destabilisation, as the radius of gyration (RoG) remained essentially constant across all systems. Notably, Baa binding decreased the solvent-accessible surface area (SASA) (12,556.45 ± 292.49 Å^2^) compared with Ba and Asp, indicating tighter molecular packing and reduced solvent exposure. This suggests enhanced conformational stabilisation of the thrombin receptor upon Baa binding, consistent with the reduced residue fluctuations (RMSF) observed in key binding regions. Hydrogen bond occupancy and temporal stability analyses further underscore Baa’s superior binding stability. The persistent hydrogen bond with Trp96 and secondary interactions with Tyr60A contributed to an average of 1–3 hydrogen bonds throughout the simulation, compared with transient bonding in Ba and moderate stability in Asp.

The physicochemical and pharmacokinetic profiles highlight 3β-acetoxybetulinic acid as a potential natural product scaffold for further structural optimisation, despite its limited oral bioavailability. Its high molecular weight and lipophilicity reflect the typical characteristics of pentacyclic triterpenoids, which contribute to poor aqueous solubility and low gastrointestinal absorption [31]. Oral delivery remains a preferred route for administering bioactive compounds; however, successful absorption through this pathway requires the drug to possess adequate aqueous solubility to traverse the gastrointestinal environment effectively [32]. Betulinic acid has been shown to exhibit poor aqueous solubility (<0.1 µg/mL) [33]. These limitations highlight the need for novel, advanced drug-delivery strategies to improve the solubility, absorption, and overall bioavailability of 3β-acetoxybetulinic acid and betulinic acid. Recent years have seen growing interest in the use of nanocarrier systems to enhance the efficiency of oral drug delivery [34]. Nanoformulation represents a novel strategy to enhance the aqueous solubility and bioavailability of betulinic acid [35]. Betulinic Acid-loaded lipid nanocarriers (BALNCs) were found to markedly enhance the drug’s solubility, bioaccessibility, and stability, achieving over 46% bioaccessibility and approximately 90% release within six hours, thereby effectively overcoming its poor solubility and bioavailability [36]. Recent studies employing self-nanoemulsifying drug delivery systems (SNEDDS) in which betulinic acid was encapsulated in fish oil demonstrated marked improvements in solubility and bioavailability compared to the free compound [37].

The moderate polar surface areas (≈60 Å^2^) and high molar refractivity values of the triterpenoids indicate balanced polarity and strong van der Waals potential, supporting their capacity for membrane interaction [38]. The superior skin permeability of the triterpenoids compared to aspirin indicates suitability for transdermal or topical delivery [39,40]. The predicted metabolic stability, selective CYP2C9 inhibition, absence of PAINS alerts, and high natural product likeness further support *3β-acetoxybetulinic acid’s* potential as a natural product scaffold for further structural modification strategies.

These results collectively indicate that 3β-acetoxybetulinic acid achieves unique, optimised binding interactions with thrombin’s aryl-binding region (S_3_ subsite), balancing hydrogen bonding with hydrophobic complementarity. This may explain its experimentally observed potency over the parent compound. From a medicinal chemistry perspective, further C-3 substitutions or C-28/30 (carboxyl) modifications [41] on the betulinic scaffold—introducing functional groups capable of forming stronger interactions with nearby residues—may yield even more potent thrombin inhibitors. Importantly, the simulations do not claim independent validation of biological activity. Rather, they mechanistically rationalise experimental findings by linking structural features to functional inhibition. The reduction in solvent-accessible surface area and residue-level fluctuations upon binding of 3β-acetoxybetulinic acid further supports a stabilising effect on the thrombin–ligand complex, which correlates with the reduced enzymatic activity observed experimentally.

## 4. Materials and Methods

The computational analyses conducted in this study were explicitly informed by previously published in vitro and ex vivo experiments demonstrating the antithrombotic, anticoagulant, and antiplatelet activities of betulinic acid and 3β-acetoxybetulinic acid [25]. These experiments employed chromogenic antithrombin assays to directly quantify thrombin enzymatic inhibition, alongside platelet aggregation assays and bleeding time models to assess functional haemostatic effects. In that study, Ba and Baa were experimentally shown to inhibit thrombin activity in a dose-dependent manner using a chromogenic substrate assay, with Baa exhibiting significantly greater inhibitory potency (IC_50_ = 1.10 ± 0.03 mg·mL^−1^) than Ba and aspirin. In addition, both compounds attenuated thrombin-induced platelet aggregation and demonstrated anticoagulant activity with comparatively reduced bleeding time in a rat tail transection model.

The chromogenic substrate assay measures thrombin’s ability to cleave a specific peptide substrate, thereby providing direct evidence of enzyme inhibition. The observed dose-dependent reduction in thrombin activity, particularly for 3β-acetoxybetulinic acid, strongly suggests interaction with thrombin’s catalytic or substrate-recognition regions. These experimentally validated outcomes provided the biological foundation for the present molecular modelling strategy.

### 4.1. Protein Structure Preparation

The crystal structure of the human thrombin receptor (PDB ID: 3BV9) was obtained from the Protein Data Bank (https://www.rcsb.org/) [42] with a resolution of 1.80 Å. The initial protein crystal structure was prepared using the Protein Preparation Wizard in Schrodinger Maestro version 2023-2. This process involved assigning bond orders, adding hydrogen atoms, and missing residues or atoms reconstructed with the Prime module using the OPLS4 force field [43]. Co-crystallised water molecules were removed, and the protonation states of ionisable residues were set to pH 7.0 using PROPKA (v 2.0). After optimising the hydrogen bond network, the entire system was minimised until the root-mean-square deviation (RMSD) of heavy atoms converged to 0.3 Å.

### 4.2. Ligand Preparation

The three-dimensional (3D) chemical structures of 3β-acetoxybetulinic acid (PubChem CID: 102379793), betulinic acid (PubChem CID: 64971), and aspirin (PubChem CID: 2244) were retrieved from the PubChem chemical database (https://pubchem.ncbi.nlm.nih.gov/) [44]. Ligand preparation was performed using the LigPrep module within the Schrödinger Suite version 2023-2 (Schrödinger, LLC, New York, NY, USA). This process included the generation of low-energy 3D conformations, protonation state assignment at physiological pH (7.2 ± 0.2) using the Epik classic tool, and energy minimisation using the OPLS4 force field [43]. The protocol was configured to produce a single conformer for each ligand, and the generation of alternative tautomers was disabled.

### 4.3. Induced-Fit Docking (IFD)—Extra Precision (XP)

To account for the conformational flexibility of both the receptor and the ligands during ligand binding, the Induced Fit Docking (IFD) protocol [45] implemented in the Schrödinger Suite was employed. The docking grid was generated by centring it on the centroid of the co-crystallised ligand within the active site. The IFD workflow was executed using a multi-step protocol. Initially, ligands were docked into the receptor using the Glide standard precision (SP) mode to generate a diverse ensemble of binding poses. During this stage, the van der Waals radii of ligand and receptor atoms were scaled by a factor of 0.50 to enhance sampling of potential binding conformations, and the Coulomb–van der Waals cutoff was adjusted to allow softer potential interactions.

For each generated ligand pose, the side chains of amino acid residues within 5.0 Å of the ligand were refined and energy-minimised using the Prime module to accommodate ligand-induced conformational changes in the binding pocket. Subsequently, the top 20 ligand poses were re-docked into their corresponding refined (induced-fit) receptor conformations using Glide extra precision (XP) mode. The resulting ligand–receptor complexes were ranked according to the IFD score, a scoring function that combines GlideScore and Prime energy contributions. For each ligand-protein complex, the pose with the lowest IFD score was selected for subsequent molecular dynamics simulations.

### 4.4. Molecular Dynamics (MD) Simulation

In this study, MD simulations were performed to investigate the ligand-binding interactions and the dynamic behaviour of thrombin in complexes with 3β-acetoxybetulinic acid, betulinic acid, and aspirin. Prior to MD trajectory production with AMBER 20, partial atomic charges for the ligands were derived using the General AMBER Force Field version 2 (GAFF2) via the *antechamber* module implemented in the AMBER 20 software package [46]. The ff14SB and GAFF2 force fields were employed to describe the protein and ligands, respectively [47]. Each complex was explicitly solvated using the TIP3P water model within a periodic boundary box at an edge distance of 12 Å, and the overall charge was neutralised by the addition of Cl- counter-ions [48]. The detailed composition of the simulated systems is provided in Table S2. A two-step energy minimisation protocol was applied to each system. First, hydrogen atoms and water molecules were minimised using 2500 steps of the steepest descent algorithm [49]. 5000 steps of the conjugate gradient method followed this without restraint. The minimised system was then heated from 10 K to 310 K using the Langevin thermostat [50] with a collision frequency of 2 ps^−1^ for 100 ps. The system was further equilibrated under the isothermal–isobaric (NPT) ensemble at 300 K and 1 atm, with pressure coupled using the Berendsen barostat [50] for 200 ps. MD production run for apo and ligand complexes were conducted for 400 ns with a 2 fs integration time step using the NPT ensemble at 1 atm and 300 K. Long-range electrostatic interactions were calculated using the Particle Mesh Ewald (PME) summation method [51], while the SHAKE algorithm was utilised to constrain all bonds involving hydrogen atoms [52]. Post-simulation trajectory analyses were conducted using the *cpptraj* module in AMBER20 to evaluate the structural stability and dynamic properties of the protein–ligand complexes [53]. The key parameters assessed from the MD trajectory included root-mean-square deviation (RMSD), root-mean-square fluctuation (RMSF), radius of gyration (RoG), and solvent-accessible surface area (SASA). Additionally, the interaction profiles between ligands and key protein residues were characterised by analysing the frequency and distribution of hydrogen bond formation throughout the simulation trajectory.

### 4.5. Binding Free Energy Calculations (MM/GBSA)

Molecular mechanics Poisson–Boltzmann surface area (MM/PBSA) and molecular mechanics Generalized Born surface area (MM/GBSA) are widely used end-point methods for estimating ligand–target binding free energies [54,55]. While MM/PBSA may better approximate absolute binding energies, MM/GBSA more reliably predicts relative affinities among related ligands and is therefore preferred for post-processing docked poses and ranking inhibitors [54,56,57,58]. Owing to its lower computational cost and consistent performance, MM/GBSA is commonly applied in structure-guided drug design [59,60]; evaluation of multiple Generalized Born models confirmed that the selected parameterization provided the most reliable affinity ranking [54,56].

The binding free energy (ΔG*_bind_*) for each complex was estimated using the MM/GBSA method, as implemented in the MMPBSA.py module of the AMBER software suite [56,61]. This approach offers a computationally efficient alternative to more rigorous alchemical free-energy methods by decomposing the binding free energy into a sum of physically distinct energy components. The binding free energy (Δ*G_bind_*) is calculated according to Equations (1) and (2):

Δ*G_bind_* = G*_complex_* − (G*_protein_* + G*_ligand_*), (1)

Δ*G_bind_* = Δ*H* – *T*Δ*S,*, (2)

where ∆*H* represents the binding enthalpy and −*T*∆*S* the entropic contribution. The entropy (−*T*∆*S*) can be estimated by using the normal mode analysis (mmpbsa_py_nabnmode) program [61] or quasi-harmonic approximation in AMBER; however, due to the high computational cost, only the enthalpic term ∆*H* was computed in this study.

In the calculations of MM-GBSA, ∆*H* was decomposed as:

ΔH = Δ*G_egb_* + Δ*G_esurf_* + Δ*E_vdw_* + Δ*E_ele_*, (3)

where Δ*E*_*vdW*_ and Δ*E*_*ele*_ correspond to van der Waals and electrostatic molecular mechanic energies, respectively. Δ*G*_*egb*_ represents the polar solvation free energy calculated using the Generalized Born (GB) solvation model [62] and Δ*G*_*esurf*_ denotes the non-polar solvation free energy estimated from the solvent accessible surface area (SASA) using:

Δ*G^esurf^* = γ × ΔSASA + β, (4)

with γ set as 0.0072 kcal/mol/Å^2^ and β set to the AMBER default constant.

Binding free-energy calculations were performed on 150 snapshots extracted from the equilibrated portion of the trajectory (100–400 ns) at 2 ns intervals, with the ionic strength set to 0.15 M.

### 4.6. In Silico Physicochemical Characterisation

The pharmacokinetic and physicochemical properties of 3β-acetoxybetulinic acid, betulinic acid, and aspirin were assessed using ADMETlab 3.0 (https://admetlab3.scbdd.com/) [63] and SwissADME (https://www.swissadme.ch/) [64,65]. The molecules were uploaded to the platforms using their PubChem SMILES representations (Table S1).

### 4.7. Data Analysis

All plots and data were generated and statistically analysed using Origin data analysis software (v 6.0) [66]. Results are presented as either averages or as mean ± standard deviation (SD). Docking complexes were visualised using DS Visualizer (v 4.2) [67], UCSF Chimera (v 1.19) [68], and Schrödinger Maestro version 2023-2.

## 5. Conclusions

This study demonstrates that 3β-acetoxybetulinic acid exhibits superior thrombin inhibition through a high-occupancy hydrogen bond with Trp96 and extensive hydrophobic interactions across the Trp215–Leu99–Ile174–Tyr60A binding network, compared to betulinic acid and aspirin. The acetoxy group substitution at C-3 is identified as a key structural determinant that enhances binding affinity and complex stability, consistent with previously observed experimental inhibition trends. It provides a starting point for further structural optimisation of the triterpenoid-based thrombin inhibitors. Despite the limited aqueous solubility and oral bioavailability typical of triterpenoids, advances in nanocarrier drug delivery systems may offer promising solutions to overcome these pharmacokinetic limitations. Future studies may focus on synthetic modifications at the C-3 and C-28/30 positions and on enhancing solubility, supported by quantum mechanics/molecular mechanics (QM/MM) and free-energy perturbation (FEP) calculations to improve activity prediction. Additionally, in vitro and in vivo pharmacokinetic evaluations, coupled with advanced formulation strategies such as nanoencapsulation or prodrug development, may improve bioavailability and therapeutic efficacy.

## Figures and Tables

**Table 1:** Molecular docking scores and key amino acid interactions of the ligands with the thrombin receptor active site residues.

Ligand	Docking Score (kcal/mol)		Interaction Residues		
	XP GScore	IFD Score	Hydrogen Bond	Hydrophobic	π–π Stacking
Baa	−8.38	−635.69	Trp96 Tyr60A	Leu99 Trp96 Val213 Trp215 Tyr60A Pro60C Cys191 Trp60D Ile174 Cys220	**-**
Ba	−8.22	−635.66	Trp96 Tyr60A	Ile174 Trp96 Tyr60A Trp215 Val213 Pro60C Cys191 Trp60D Leu99	**-**
Asp	−5.49	−631.05	Gly216 Ser195	Leu99 Tyr60A Trp60D Cys191 Val213 Trp215	His57 Tyr60A Trp60D

**Table 2:** Comparative physicochemical, pharmacokinetic, and medicinal chemistry properties of 3β-acetoxybetulinic acid, betulinic acid, and aspirin.

	Baa	Ba	Asp
Physicochemical Properties			
Molecular weight (MW) (g/mol)	498.37	456.36	180.04
Number of H-bond Donor (HBD)	1	2	1
Number of H-bond Acceptor (HBA)	4	3	4
Number of Rotatable Bonds	4	2	3
Topological Polar Surface Area (TPSA) (Å^2^)	63.60	57.53	63.60
^#^ Molar Refractivity	146.65	136.91	44.90
Lipophilicity and Solubility			
Partition coefficient (Log P_o/w_)	4.65	4.25	1.17
Solubility coefficient (LogS)	−4.97	−4.95	−1.55
Pharmacokinetics			
^#^ GI absorption	Low	Low	High
^#^ BBB permeation	No	No	Yes
^#^ CYP1A2 inhibitor	No	No	No
^#^ CYP2C19 inhibitor	No	No	No
^#^ CYP2C9 inhibitor	Yes	No	No
^#^ CYP2D6 inhibitor	No	No	No
^#^ CYP3A4 inhibitor	No	No	No
^#^ *P*-gp Substrate	No	No	No
^#^ Log K_p_ (skin permeation) (cm/s)	−3.11	−3.26	−6.55
Caco-2 Permeability	−5.21	−5.35	−4.99
CL_plasma_ (ml/min/kg)	2.44	6.35	2.77
T_1/2_	0.97	0.79	0.82
Medicinal Chemistry			
PAINS (alert)	0	0	0
Synthetic Accessibility (SA) score	Easy	Easy	Easy
Natural Product Likeness Score (NPscore)	3.01	3.07	0.12

CaCo-2—Absorption in human colon adenocarcinoma cells; ^#^ Parameters derived from SwissADME.

**Table 3:** Hydrogen bond occupancy between 3β-acetoxybetulinic acid, betulinic acid, aspirin and key binding site amino acids of thrombin.

Complex	H-Bond Acceptor	H-Bond Donor	Occupancy (%)	Average Distance (Å)	Average Angle (°)
Baa	Trp96-O	Lig248-O3…H2	95.51	2.70	159
	Lig248-O1	Tyr60A-OH…HH	25.64	2.76	162
Ba	Trp96-O	Lig248-O3…H2	4.73	2.79	160
	Lig248-O3	Ser195-OG…HG	2.82	2.78	163
	Lig248-O1	Trp148-NE1…HE1	1.93	2.87	153
	Ser214-O	Lig248-O3…H2	1.84	2.78	161
	Gly216-O	Lig248-O3…H2	1.83	2.77	157
Asp	Lig248-O1	Leu99-N…H	39.88	2.88	161
	Trp215-O	Lig248-O2…H1	32.26	2.75	160
	Lig248-O2	Leu99-N…H	2.80	2.91	162
	Lig248-O1	Gly193-N…H	1.50	2.89	158

**Table 4:** Predicted MM/GBSA free energies (kcal/mol) analysis of three studied compounds in complex with thrombin: 3β-acetoxybetulinic acid, betulinic acid, and the control aspirin.

Energy Components	Complexes		
	Baa	Ba	Asp
Van der Waal energy (Δ_vDWaals_)	−35.26 ± 2.79	−30.65 ± 7.09	−22.62 ± 2.62
Electrostatic energy (Δ_ELECT_)	−11.30 ± 4.14	−5.49 ± 5.51	−20.02 ± 4.99
Polar solvation energy (ΔE_GB_)	21.36 ± 2.94	18.79 ± 5.21	27.20 ± 3.55
Non-polar solvation energy (ΔE_SURF_)	−4.38 ± 0.38	−3.59 ± 0.88	−3.44 ± 0.22
Net gas phase energy (ΔG_GAS_)	−46.57 ± 4.74	−36.14 ± 9.02	−42.63 ± 6.38
Net solvation energy (ΔG_SOLV_)	16.98 ± 2.85	15.20 ± 4.84	23.76 ± 3.43
ΔG_TOTAL_	−29.58 ± 2.97	−20.94 ± 5.81	−18.87 ± 4.18

**Table 5:** Per-residue free energy (ΔG_total_) contributions of hotspot residues involved in the binding of 3β-acetoxybetulinic acid, betulinic acid, and aspirin to the target protein. Residues that contributed significantly to ΔG_Total_ (ΔG_Total_ > 1.00 kcal/mol) were defined as hotspot residues.

	Baa	Ba	Asp
	ΔG_Total_	ΔG_Total_	ΔG_Total_
His57	−0.33 ± 0.29	−0.25 ± 0.42	-
Tyr60A	−1.31 ± 1.07	−0.41 ± 0.42	−0.26 ± 0.18
Pro60C	-	−0.29 ± 0.42	-
Trp60D	−0.57 ± 0.36	−1.60 ± 0.98	-
Trp96	−1.65 ± 0.47	-	-
Arg97	−0.33 ± 0.20	-	-
Asn98	−1.02 ± 0.29	−0.42 ± 0.54	−1.45 ± 0.38
Leu99	−1.57 ± 0.35	−0.82 ± 0.71	−2.00 ± 0.44
Trp148	-	−0.70 ± 1.18	-
Thr172	-	-	−0.26 ± 0.22
Ile174	−1.67 ± 0.36	−0.68 ± 0.88	−1.84 ± 0.47
Ile176	-	-	−0.61 ± 0.32
Met180	-	-	−0.29 ± 0.21
Ala190	-	−0.21 ± 0.32	-
Cys191	-	−0.26 ± 0.30	-
Glu192	−0.48 ± 0.60	−0.43 ± 0.62	-
Gly193	−0.29 ± 0.32	-	-
Trp215	−2.03 ± 0.48	−1.12 ± 0.74	−1.73 ± 1.32
Gly216	-	-	−0.24 ± 0.49
Phe227	-	-	−0.79 ± 0.30
Ligand289	−19.99 ± 1.59	−15.09 ± 3.98	−9.38 ± 2.21

## Data Availability

The data are contained within the article.

## Supplementary Materials

The following supporting information can be downloaded at https://www.mdpi.com/article/10.3390/molecules31050848/s1, Table S1. The Canonical SMILES strings of compounds used for physicochemical and ADME property predictions. Table S2. Composition of the simulated systems, including numbers of water molecules, ions, ligand atoms, residues, and total atoms.

## Abbreviations

The following abbreviations are used in this manuscript:

Asp	Aspirin
Ba	Betulinic Acid
Baa	3β-Acetoxybetulinic Acid
BALNCs	Betulinic Acid-Loaded Lipid Nanocarriers
MD	Molecular Dynamics
MM/GBSA	Molecular Mechanics/Generalised Born Surface Area
PC	Principal Components
RMSD	Root Mean Square Deviation
RMSF	Root Mean Square Fluctuation
RoG	Radius of Gyration
SASA	Solvent-Accessible Surface Area
SNEDDS	Self-Nanoemulsifying Drug Delivery Systems
