# Supplementary material for: Computational and Molecular Dynamics Insights into the Antithrombotic Mechanism of Triterpenes Derived from Melaleuca bracteata var. Revolution Gold (Myrtaceae)
Source: Molecules. 2026 Mar 3;31(5):848. doi: 10.3390/molecules31050848 (PMC12986523; doi:10.3390/molecules31050848)
Supplement: Supplementary file 1 [file molecules-31-00848-s001.zip › molecules-4147475-supplementary.pdf]

Supplementary Materials

Computational and Molecular Dynamics Insights into the Antithrombotic Mechanism of triterpenes derived from *Melaleuca bracteate* var. Revolution Gold (Myrtaceae)

Patrick Appiah-Kubi[1], Foluso Oluwagbemiga Osunsanmi[2*], Andrew Rowland Opoku[2], Ashona Singh[1*]

Table S1. The Canonical SMILES strings of compounds used for physicochemical and ADME property predictions.

	Molecular Formula	Canonical SMILES
3β-acetoxybetulinic acid (Baa)	C32H50O4	CC(=C)[C@@H]1CC[C@]2([C@H]1[C@H]3CC[C@@H]4[C@]5(CCC(C([C@@H]5CC[C@]4([C@@]3(CC2)C)C)(C)C)OC(=O)C)C)C(=O)O
Betulinic acid (Ba)	C30H48O3	CC(=C)[C@@H]1CC[C@]2([C@H]1[C@H]3CC[C@@H]4[C@]5(CC[C@@H](C([C@@H]5CC[C@]4([C@@]3(CC2)C)C)(C)C)O)C)C(=O)O
Aspirin (Asp)	$C_9H_8O_4$	CC(=O)OC1=CC=CC=C1C(=O)O

Table S2. Composition of the simulated systems, including numbers of water molecules, ions, ligand atoms, residues, and total atoms.

Simulating Systems	Number of Water		Counter Ions		Number of Ligand atoms	Number of Residues atoms (288 residues)	Total Number of Simulating Atoms
	Molecules	Atoms	Cl-	Na+			
Apo	12276	36828	2	-	-	4640	41470
Asp	12369	37107	2	-	21	4640	41770
Ba	12254	36762	2	-	81	4640	41485
Baa	12255	36765	2	-	86	4640	41493
